# Different associations between intelligence and social cognition in children with and without autism spectrum disorders

**DOI:** 10.1371/journal.pone.0235380

**Published:** 2020-08-21

**Authors:** Tetsu Hirosawa, Keiko Kontani, Mina Fukai, Masafumi Kameya, Daiki Soma, Shoryoku Hino, Tatsuru Kitamura, Chiaki Hasegawa, Kyung-min An, Tetsuya Takahashi, Yuko Yoshimura, Mitsuru Kikuchi

**Affiliations:** 1 Department of Psychiatry and Neurobiology, Graduate School of Medical Science, Kanazawa University, Kanazawa, Japan; 2 Research Center for Child Mental Development, Kanazawa University, Kanazawa, Japan; 3 Department of Neuropsychiatry, Ishikawa Prefectural Takamatsu Hospital, Ishikawa, Japan; 4 Faculty of Education, Institute of Human and Social Sciences, Kanazawa University, Kanazawa, Japan; Virginia Polytechnic Institute and State University, UNITED STATES

## Abstract

Autism spectrum disorders (ASD) are characterized by impaired social cognition and communication. In addition to social impairment, individuals with ASD often have intellectual disability. Intelligence is known to influence the phenotypic presentation of ASD. Nevertheless, the relation between intelligence and social reciprocity in people with ASD remains unclear, especially in childhood. To elucidate this relation, we analyzed 56 typically developing children (35 male, 21 female, aged 60–91 months) and 46 children with ASD (35 male, 11 female, aged 60–98 months) from university and affiliated hospitals. Their cognitive function was evaluated using the Kaufman Assessment Battery for Children. Their social cognition was assessed using the Social Responsiveness Scale. We used linear regression models to ascertain whether the associations between intelligence and social cognition of typically developing children and children with ASD are significantly different. Among the children with ASD, scores on the Kaufman Assessment Battery for Children correlated significantly with social cognition, indicating that higher intelligence is associated with better social cognition. For typically developing children, however, no significant correlation was found. One explanation might be that children with ASD fully use general intelligence for successful learning in social cognition, although extensive use of intelligence might not be necessary for TD children. Alternatively, autistic impairment in social cognition can be compensated by intelligence despite a persistent deficit in social cognition. In either case, when using the SRS as a quantitative phenotype measure for ASD, the influence of intelligence must be considered.

## Introduction

Numerous and diverse difficulties driven by social impairment are experienced by individuals with autism spectrum disorder (ASD), a neurodevelopmental disorder associated with repetitive behaviors and characterized by impaired social cognition and communication [1]. For example, social impairment leads people with ASD to experience difficulties in education, employment, and in severe cases, independent living [2–5]. Moreover, it is noteworthy that severe social impairment in childhood strongly predicts those factors later in their adulthood [6], which emphasizes the importance of understanding factors associated with childhood social impairment.

Many individuals with ASD have intellectual disability (ID) in addition to social impairment [7]. Although shared etiologies are largely unknown, some researchers have inferred shared genetic risk factors [8] and prenatal risk factors such as exposure to infection [9], medications [10], and preterm delivery [11]. Furthermore, earlier reports describe that intelligence and autistic symptomatology might not be mutually independent. For instance, repetitive and restrictive behaviors (RRBs) have been shown to correlate with a lower nonverbal developmental quotient [12–16], with the sole exception of a study by Joseph et al. which found no significant relation [17]. As another illustrative instance, Bishop et al. explored the relation between nonverbal IQ and RRBs in 830 children with ASD and reported that the prevalence of most RRBs (e.g. repetitive use of objects, and hand and finger mannerisms) is negatively associated with nonverbal IQ. Conversely, the association was positive for RBBs of certain types (e.g., circumscribed interests) [16].

In addition, the relation between social cognition and intelligence in ASD or PDDNOS has been debated since the 1990s [18–20]. For instance, Buitelaar et al. examined 60 children: 20 children with autism; 20 with pervasive developmental disorder, not otherwise specified (PDDNOS); and 20 children under psychiatric control. Performance IQ and verbal memory were found to be strong predictors of performance on emotion recognition tasks. A significant positive correlation between performance IQ and second-order ToM was also reported, although the effect was not found to be significant for first-order ToM. They reported relationships throughout the sample, but also reported that the coefficient patterns did not differ markedly across the three diagnostic groups [18]. Subsequent studies of ASD and PDDNOS populations using various measures of social cognition almost invariably found significant association between better social cognition and higher intelligence [21–29]. For instance, Livingston et al. measured ASD symptoms using ADOS-G and compared children with ASD having milder ASD symptoms despite poor ToM performance, with children who had severer ASD symptoms and poor ToM performance. They reported the former group as having higher intelligence [29]. One exception is a study conducted by Muller et al. They evaluated participants’ social cognition using a Movie for the Assessment of Social Cognition (MASC), in which the participants inferred film characters’ mental states including emotions, thoughts, and intentions. Based on the MASC results, Muller et al. reported non-significant association between intelligence and social cognition [30].

Some studies have explored the relation between social cognition and intelligence in TD populations [22, 25, 28, 30–33]. Unlike those in ASD populations, some reports have described results showing non-significant correlation [22, 30–32]. Others have reported significant correlation [25, 28, 33]. Mohn et al. examined 250 healthy adults and reported non-significant association between IQ and social cognition, as measured using the Mayer–Salovey–Caruso Emotional Intelligence Test (MSCEIT) [31]. Farrelly et al. reported significant association between social cognition measured using the same battery (MSCEIT) and crystallized intelligence, as measured using the Gf/Gc Quickie Test Battery [33].

More generally, some other studies examined how autistic social impairment and intelligence are related. A few studies have examined the association between intelligence and social communication or motivation. Those studies examined ASD populations and found significant correlation between intelligence and social communication [34–36]. Qualls et al. examined adolescents with ASD and reported positive association between scores of the Social Communication Questionnaire (SCQ; [37]) and IQ [34]. To assess a general population, one study conducted with more than 5000 participants [38] revealed that higher verbal IQ has a significant association with better social communication, as measured by Social Communication Disorders Checklist and Children’s Communication Checklist, only in female participants. For social motivation, two studies have been conducted. Dawson et al. reported that lower IQ is related to lower social motivation, as measured by Broader Phenotype Autism Symptom Scale, in an ASD population [39]. Dubey et al. assessed social motivation by their “choose-a-movie paradigm measures” and reported that correlation was not significant for a combined population including both TD and ASD [40].

The Social Responsiveness Scale (SRS) [41] is a widely used rating scale for autistic symptomatology. It measures the severity of autistic symptomatology as a quantitative trait among children with ASD and among TD children. Actually, SRS comprises five sub-scales of the autistic mannerism sub-scale and four components of social reciprocity: receptive (social awareness sub-scale), cognitive (social cognition sub-scale), expressive (social communication sub-scale), and motivational (social motivation sub-scale) subdomains [41]. Those autistic traits and symptoms measured using SRS are distributed continuously, in ASD populations and in the general population [42]. Therefore, SRS is particularly useful for characterizing milder social impairment such as that which lies at the boundary between the normal population distribution and clinical-level affectation. In this sense, SRS might be suitable for examining the association between potentially milder autistic symptoms and intelligence. According to this framework, four studies have been conducted [43–46]. Hus et al. recruited 2,368 individuals with ASD and 1,913 unaffected siblings. Subsequently, they assessed factors influencing the SRS raw scores. They reported the respective associations of higher SRS scores with greater non-ASD behavior difficulties, higher age, more impaired language, and lower non-verbal IQ in an ASD population. It is particularly interesting that non-verbal IQ effects on the SRS raw score were not found to be significant for unaffected siblings [45]. Three other recent studies specifically examined the sub-scales of SRS. The results are inconsistent. Rodgers et al. examined the association in a sample of children with ASD using the SRS-Second Edition (SRS-2; [47]) and the Wechsler Intelligence Scale for Children-4th Edition (WISC-IV; [48]). They reported that lower IQ is significantly associated with higher scores in the social cognition sub-scale (i.e., greater difficulties with social cognition) for girls only. No significant correlation was found for any other sub-scale [43]. Torske et al. assessed children with ASD or PDDNOS using the SRS [41]), age-appropriate Wechsler tests of intelligence, and Behavior Rating Inventory of Executive Function (BRIEF). They reported that IQ did not correlate to any sub-scale [44]. Chouinard et al. examined adolescent participants with and without ASD. They reported that higher IQ was correlated with a better social communication sub-scale of SRS [46].

Overall, studies of ASD subjects have tended to find significant association between intelligence and subdomains of social reciprocity. Studies of TD subjects have tended to find non-significant association. The relation in ASD children might differ from those in TD children. Actually, only one report describes a study examining this subject [22]. For that study, Kim et al. examined adolescent participants with and without ASD using a facial emotion recognition task in which participants identify six emotions expressed by avatars: happiness, fear, anger, disgust, sadness, and surprise. They reported that full scale IQ was positively correlated with accuracy of happy expression identification only in the ASD sample. Moreover, the group difference of the correlations was significant.

The study described herein was conducted to extend findings reported by Kim et al. [22] and those we described above to assess differences in the relations between intelligence and subdomains of social reciprocity in children with ASD and those in TD children. We narrowed the age range of the participants because social ability in childhood might influence the development of intelligence [49]. Consequently, the relation found for age-inhomogeneous participants might be unreliable. We hypothesize that the relations between intelligence and social reciprocity differ in children with ASD and in TD children. More specifically, higher intelligence is associated with better social awareness, social cognition, social communication, and social motivation only in children with ASD.

## Materials and methods

### Participants

From Kanazawa University and affiliated hospitals, we recruited 56 TD children (35 male, 21 female, aged 60–91 months) and 50 children with ASD (38 male, 12 female, aged 60–98 months). Four children with ASD were unable to complete the psychometric evaluation because of their severe psychomotor agitation. For those children, the experimenter decided to halt the evaluation. Their data were excluded from statistical analyses. Consequently, we analyzed 56 TD children and 46 children with ASD (35 male, 11 female, aged 60–98 months).

The ASD diagnosis was made according to the Diagnostic and Statistical Manual of Mental Disorders, Fourth edition (DSM-IV) [1] using the Diagnostic Interview for Social and Communication Disorders (DISCO) [50] or the Autism Diagnostic Observation Schedule–Generic (ADOS-G) [51]. The exclusion criteria were (1) blindness, (2) deafness, (3) any other neuropsychiatric disorder including epilepsy, and (4) ongoing medication. Written informed consent was obtained from parents before participation by the children. The Ethics Committee of Kanazawa University Hospital approved the methods and procedures, all of which were conducted in accordance with the Declaration of Helsinki.

We are continually recruiting participants as part of a single large project (Bambi plan, http://bambiplan.w3.kanazawa-u.ac.jp/pdf/jusen_english.pdf). Some participants overlap with those of our earlier study [52]. However, the results do not. In addition, the emphases of that earlier study differed from those of the present study.

### Assessment of intelligence

For this study, we assessed the intelligence of the participants using the Japanese version of the Kaufman Assessment Battery for Children (K-ABC) [53]. In K-ABC, the set of skills for problem-solving abilities is interpreted as intelligence. Knowledge of facts is defined as achievement. In this sense, K-ABC was developed to distinguish intelligence from knowledge [53, 54]. In K-ABC, intelligence is measured on the Sequential Processing Scale and the Simultaneous Processing Scale. The Mental Processing Scale (MPS), a unification of those two scales, is intended as a measure of total intelligence in the assessment battery [54]. On the Sequential Processing Scale, the child must solve problems in a serial, stepwise manner. The subtests included in this scale are *Hand Movements*, *Number Recall*, and *Word Order*. For the Simultaneous Processing Scale, the child must integrate many stimuli simultaneously to solve problems. The subtests included in this scale are *Magic Window*, *Face Recognition*, *Gestalt Closure*, *Triangles*, *Matrix Analogies*, *Spatial Memory*, and *Photo Series*. The Achievement Scale (ACH) provides an estimate of earlier learning. It reflects the effectiveness by which a child applies intelligence in real-life situations [55]. The subtests for ACH are *Expressive Vocabulary*, *Faces and Places*, *Arithmetic*, *Riddles*, *Reading/Decoding*, and *Reading/Understanding*. Kamphaus et al. reported that aCH includes measures of what have traditionally been identified as verbal intelligence (i.e., verbal concept formation and vocabulary) and acquired general information or school skills (arithmetic, letter and word reading, and word and sentence comprehension). We described details of the K-ABC sub-scales in S1 File. Some subtests are given at all ages. Others are given to selected age groups. Scores are provided as age-adjusted standardized scores, normalized to have mean of 100 and standard deviation of 15.

Haddad and Naglieri asserted that K-ABC differs from the Wechsler Intelligence Scale for Children–Revised (WISC-R; [56]): WISC splits intelligence by verbal vs. non-verbal, but K-ABC measures intelligence based on the processing style necessary to solve tasks; moreover, it separates that assessment from achievement. Reported correlations of MPS with Full Scale IQ measured using the WISC-R tend to be moderate (0.62–0.76) [53]. Those results imply that intelligence measured using the K-ABC resembles that measured using the WISC-R, but they are not identical. In fact, they have their own unique characteristics. The unique characteristics of K-ABC might be a result derived from numerous factors including the following: (i) Limited verbalization is required of the child. In fact, K-ABC uses a minimal amount of verbal involvement in measuring intellectual processing [54]. (ii) The use of “teaching” items helps to ensure that all children understand what is expected of them for each task. (iii) The inclusion of a wider variety of tasks in the K-ABC than the variety found in WISC (e.g., *Gestalt Closure*, *Face Recognition*, *Word Order*). K-ABC is expected to be a good test battery for Children with ASD because they might have good intelligence irrespective of social or language impairment.

### Assessment of social reciprocity

We assessed participants’ social reciprocity using SRS [41]: a 65-item rating scale that measures sociality and autistic mannerisms as a quantitative trait for TD children and for children who are clinically affected by autism spectrum conditions. It measures the social awareness sub-scale, social cognition sub-scale, social communication sub-scale, social motivation sub-scale, and autistic mannerisms sub-scale to generate a single measure. In both groups, a parent of each participant filled out the SRS. We used gender-normed T scores (SRS-T) of each sub-scale [57]. Higher scores represent greater difficulties with social cognition.

Although the validity of self-ratings of children is still under study, the SRS can be completed by a parent, a teacher, or another adult informant. In this way, it involves ratings of children in their natural social contexts and reflects what has been observed consistently over weeks or months of time rather than merely reflecting results of a single clinical or laboratory observation [41]. Therefore, it capitalizes on both direct observation and on the accumulated history of behaviors observed by the informant over time. By virtue of this characteristic, SRS reflects social abilities appearing not in only one-to-one real-world communication but also in one-to-many or many-to-many real-world communication, rather than those appearing in one-to-one communications or virtual communications in an examination room. For that reason, good agreement has been reported for comparison with other parents or teacher reported ASD-directed behavior assessments (e.g., SCQ [37, 58, 59]), Children’s Communication Checklist ([58–60]), and Social and Communication Disorders Checklist [61]). The SRS scores are also known to exhibit high inter-rater reliability [62] and are known to be distributed continuously in a general population [42].

### Statistical analysis

We tested differences in age and scores in K-ABC and SRS between TD and ASD using Student *t*-tests. Sex difference was tested using chi-square tests.

#### Statistical analysis–Regression analysis

Before we applied linear regression, we verified that our data meet the assumptions for regression analysis. Specifically, we used standard methods to verify linearity, normality, homogeneity of variance, model specifications, influence, and collinearity. In some cases, the assumption of homogeneity was violated. Therefore, we used heteroscedasticity-robust standard errors [63].

First, to elucidate different relations between intelligence and social reciprocity in children with ASD from those in TD children, we applied linear regression models to predict the four SRS sub-scales (i.e., social awareness sub-scale, social cognition sub-scale, social communication sub-scale, social motivation sub-scale), respectively, based on the participants’ condition (i.e., ASD or TD), K-ABC sub-scales (i.e., MPS or ACH), age, and sex as well as their interaction. We incorporated age and sex in the models because of their possible influences on SRS scores [57, 64]. We did not examine relations between cognitive performance and the diagnostic group for the autistic mannerism sub-scale because our emphasis was to ascertain different relations between social reciprocity and intelligence.

Second, if a significant interaction effect was found, then we applied post-hoc analysis to elucidate the relation between SRS sub-scales and MPS or ACH further. We predicted the sub-scale based on MPS or ACH controlling for age and sex, respectively, in each condition (i.e., ASD or TD) using linear regression models.

#### Statistical analysis–Coarsened exact matching

The results of simple regression analysis alone can be misleading if patient characteristics are very different between the conditions. In such cases, two options are available to address the imbalances: multiple regression and matching. For the detection of class effects, a regression model is often a more powerful tool than matching [65]. However, matching might be preferred in some cases (e.g., when the linearity assumption fails). To investigate the relations between social cognition and general intelligence or achievement further, we matched the groups in terms of MPS and ACH scores, age, and sex.

We improved the balance by coarsened exact matching (CEM). Then we performed adjusted regression analysis. In the CEM algorithm, we temporarily coarsen (or categorize) each variable based on its distribution or on natural or intuitive divisions. Each participant is then assigned to one of a specified set of strata in which the participant characteristics are exactly matched on a set of coarsened, or categorized variables. A weighting variable (CEM-weight) is generated to equalize the number of treated and control cases in one stratum. It is used for subsequent regression analysis [66].

We matched ASD and TD groups on MPS and ACH, age, and sex. As a binning algorithm, we used Scott’s rule [66]. This report describes the degree of imbalance between the two datasets before and after matching by measuring the multivariate L1 distance. The L1 distance, which is a value between zero and one, represents how two groups are balanced in terms of matched variables. Smaller values signify better balance. Larger values signify imbalance.

In subsequent regression analysis, we predict social cognition sub-scale based on the condition, and MPS or ACH as well as their interaction with CEM-weight for weighting. If a significant interaction effect was found, then we predicted social cognition sub-scale based respectively on MPS or ACH, in each condition.

For all statistical analyses, we inferred significance for *P* < .05. All statistical analyses were conducted using software (Stata ver. 15.0; Stata Corp., College Station, TX, USA).

## Results

We found significant differences in age [*t*(100) = -2.96, *p*< .01], Mental Processing Scale [*t*(100) = 3.85, *p* < .01], Achievement Scale [*t*(100) = 5.05, *p* < .01, and SRS total score [*t*(100) = -11.0, *p*< .01], social awareness sub-scale [*t*(100) = -7.64, *p*< .01], social cognition sub-scale [*t*(100) = -9.33, *p*< .01], social communication sub-scale [*t*(100) = -9.71, *p*< .01], social motivation sub-scale [*t*(100) = -5.77, *p*< .01] between TD and ASD groups. Table 1 presents results. Children with ASD are older. They also had lower K-ABC and higher SRS sub-scale scores.

**Table 1:** Characteristics of the participants.

	TD	ASD	χ2	*t*	*p*
*N*	56	46			
Sex (% Male)^†^	63%	76%	2.17		.141
Age in months^‡^	68.5 (6.8)	73.1 (10.3)		-2.96	< .001
K-ABC scores					
Mental Processing scale^‡^	107.4 (13.9)	91.5 (17.7)		5.05	< .001
Achievement scale^‡^	103.9 (14.2)	92.2 (16.3)		3.85	< .001
SRS-T scores					
Total^‡^	46.9 (6.9)	68.9 (12.9)		-11	< .001
social awareness sub-scale^‡^	47.4 (9.4)	61.9 (9.8)		-7.64	< .001
social cognition sub-scale^‡^	49.7 (8.2)	69.6 (13.2)		-9.33	< .001
social communication sub-scale^‡^	45.7 (6.8)	66.1 (13.8)		-9.71	< .001
social motivation sub-scale^‡^	50.3 (6.8)	63.0 (14.5)		-5.77	< .001

Numbers are mean (standard deviation) or counts.

‡ Student *t*-test.

† Chi-square test.

K-ABC, Kaufman Assessment Battery for Children.

### Group difference in associations between K-ABC sub-scales and SRS sub-scales–regression analysis

Significant interaction between condition and K-ABC sub-scales was found only in the model for the social cognition sub-scale. For the social cognition sub-scale, in the model using MPS, the interaction effect was found to be significant [*t*(96) = -2.33, *p* = .02]. The main effect of the condition [*t*(96) = 3.57, *p* < .01] was also found to be significant. No other factor was found to be significant. Similarly, in the model using ACH, the interaction effect was significant [*t*(96) = -2.19, *p* = .04]. A main effect of the condition [*t*(96) = 3.54, *p* < .01] was also significant. The results are presented in Table 2. The results in the models for other SRS sub-scales are presented in S1 Table in S1 File.

**Table 2:** Association between social cognition sub-scale and K-ABC sub-scales controlling for age and sex.

vs. social cognition sub-scale	Coeff.	SE	β	*t*	*P*>*t*	95% CI		F	*p*	*R*^2^
MPS	0.05	0.10	0.06	0.48	0.63	-0.15	0.24	22.16	< .001	.53
Condition (ASD, 1; TD, 0)	49.61	13.91	1.70	3.57	<0.01	22.00	77.21			
Condition × MPS	-0.32	0.14	-1.04	-2.33	0.02	-0.60	-0.05			
Sex	-2.56	2.36	-0.08	-1.08	0.28	-7.25	2.13			
Age (months)	0.21	0.14	0.13	1.50	0.14	-0.07	0.49			
vs. social cognition sub-scale	Coeff.	SE	β	*t*	*P*>*t*	95% CI		F	*p*	*R*^2^
ACH	-0.02	0.08	-0.02	-0.20	0.84	-0.17	0.14	23.17	< .001	.53
Condition (ASD, 1; TD, 0)	47.52	13.42	1.62	3.54	<0.01	20.88	74.17			
Condition × ACH	-0.31	0.14	-0.99	-2.19	0.03	-0.58	-0.03			
Sex	-2.47	2.29	-0.08	-1.08	0.28	-7.00	2.07			
Age (months)	0.15	0.14	0.09	1.07	0.29	-0.12	0.41			

MPS, mental processing scale; ACH, achievement scale; Coeff, regression coefficient; SE, robust standard error; CI, confidence interval; ASD, autism spectrum disorder; TD, typically developed controls.

**Fig 1:**
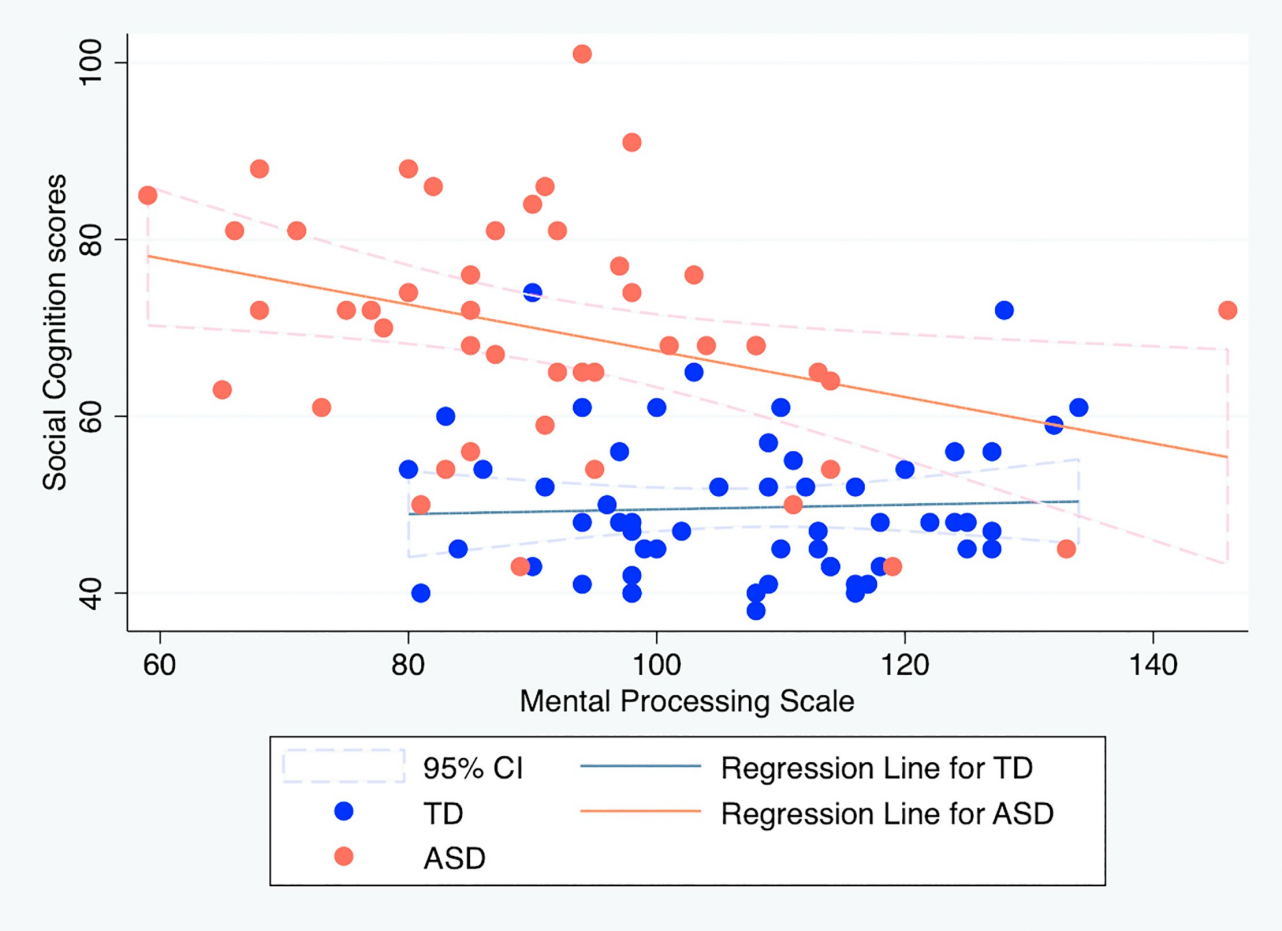
Relation between social cognition sub-scale scores and achievement scale scores on the Kaufman Assessment Battery for Children. TD, typically developing children; ASD, children with autism spectrum disorder.

**Fig 2:**
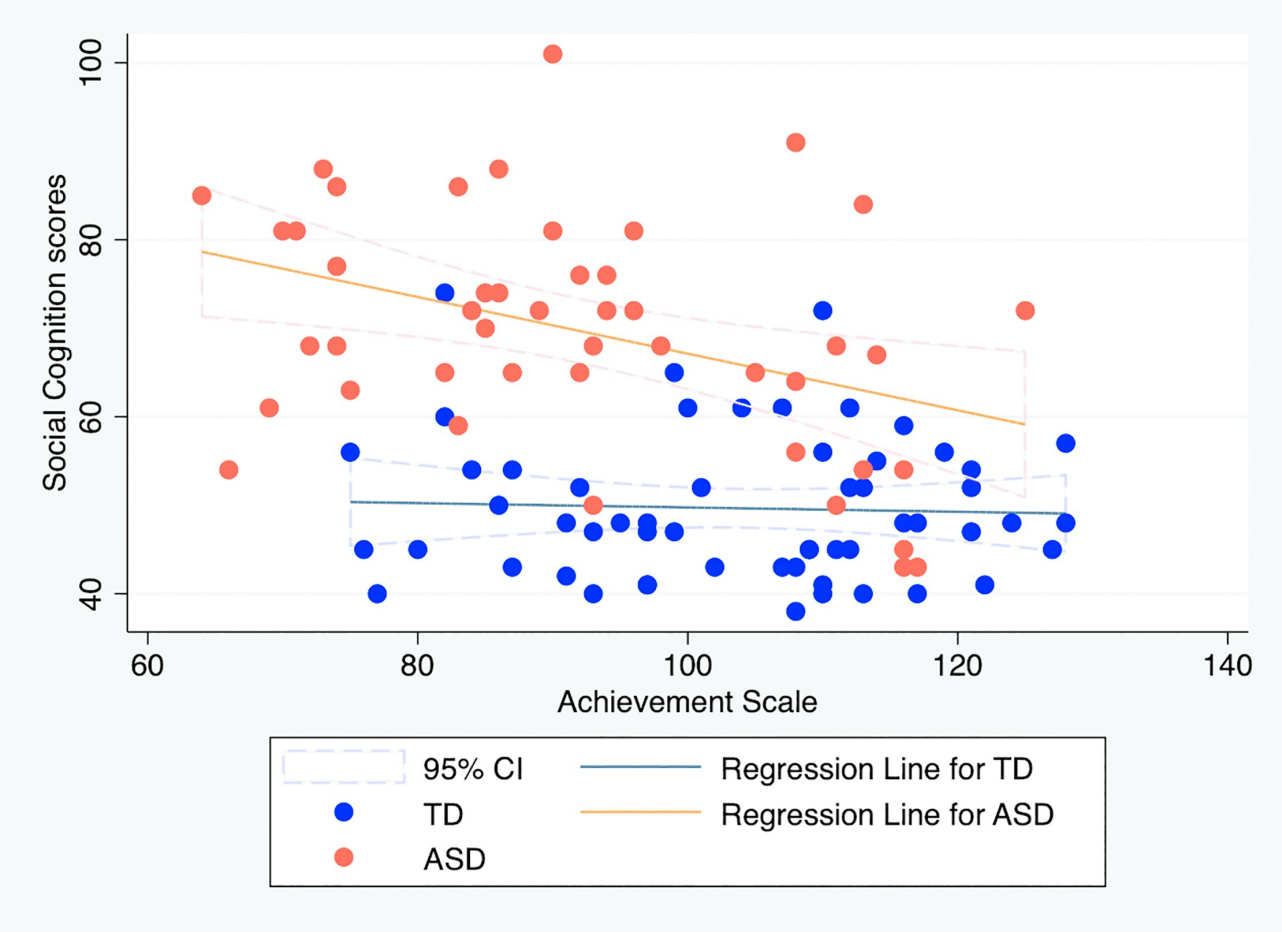
Relation between social cognition sub-scale scores and mental processing scale scores on the Kaufman Assessment Battery for Children. TD, typically developing children; ASD, children with autism spectrum disorder.

For reference purposes, we provide scatter plots and draw regression lines to visualize the associations between social cognition sub-scale and each K-ABC scale. For them, simple linear regressions were calculated to predict social cognition sub-scale based on MPS or ACH in each group (Figs 1 and 2). In the model using MPS, the main effect of the condition [*t*(98) = 3.43, *p* < .01] was found to be significant. The interaction effect was also significant [*t*(98) = -2.09, *p* = .04]. No other factor was found to be significant. In the model using ACH, the main effect of the condition [*t*(98) = 3.36, *p* < .01] was found to be significant. The interaction effect was also significant [*t*(98) = -2.03, *p* = .04]. No other factor was found to be significant. The results are presented in S2 Table in S1 File.

### Association between social cognition sub-scale and K-ABC scores in respective groups–regression analysis

To elucidate the relation between the social cognition sub-scale and general intelligence, or achievement, we applied linear regression to predict the social cognition sub-scale based on MPS or ACH, age, and sex in each group.

For TD children, in the model using MPS, a significant main effect of age was found [*t*(50) = -2.19, *p* = .03]. No other factor was found to be significant. A significant main effect of age was also significant [*t*(50) = -2.20, *p* = .03] in the model using ACH.

For children with ASD, in the model using MPS, significant main effects were found for MPS [*t*(42) = −3.68, *p* < .01], and sex [*t*(42) = -2.74, *p* < .01], and age [*t*(42) = 3.11, *p* < .01]. No other factor was found to be significant. Similar significant effects were identified in the model using ACH. Those results indicate that higher intelligence and achievement, younger age, and being male are associated with better social cognition in children with ASD. The results are presented in Table 3.

**Table 3:** Association between social cognition sub-scale and K-ABC sub-scales for respective groups controlling for age and sex.

vs. social cognition sub-scale	Coeff.	SE	β	*t*	*P*>*t*	95% CI		F	*P*	*R*^2^
TD										
MPS	0.01	0.09	0.02	0.15	0.88	-0.16	0.19	1.94	.13	.05
Sex	1.07	2.54	0.06	0.42	0.68	-4.03	6.16			
Age (months)	-0.26	0.12	-0.21	-2.19	0.03	-0.49	-0.02			
ACH	-0.03	0.08	-0.05	-0.40	0.69	-0.18	0.12	1.90	.14	.06
Sex	1.20	2.47	0.07	0.49	0.63	-3.76	6.15			
Age (months)	-0.26	0.12	-0.22	-2.20	0.03	-0.49	-0.02			
vs. social cognition sub-scale	Coeff.	SE	β	*t*	*P*>*t*	95% CI		F	*P*	*R*^2^
ASD										
MPS	-0.33	0.09	-0.45	-3.68	<0.01	-0.52	-0.15	9.22	< .001	.36
Sex	-11.34	4.14	-0.37	-2.74	0.01	-19.70	-2.98			
Age (months)	0.56	0.18	0.43	3.11	<0.01	0.20	0.92			
ACH	-0.35	0.10	-0.43	-3.55	<0.01	-0.55	-0.15	10.21	< .001	.34
Sex	-10.79	3.99	-0.35	-2.71	0.01	-18.84	-2.74			
Age (months)	0.45	0.18	0.35	2.48	0.02	0.08	0.82			

MPS, mental processing scale; ACH, achievement scale; SRS, social responsiveness scale.

Coeff., regression coefficient; SE, robust standard error; CI, confidence interval; ASD, autism spectrum disorder; TD, typically developed controls.

### Association between the social cognition sub-scale and K-ABC scores in respective groups, restricted to a sample with MPS of 90 or higher–regression analysis

Potentially, low general intelligence hinders social cognitive scores, but once a certain threshold is reached, increased intelligence does not provide any additional performance. Therefore, as an exploratory analysis, we excluded 27 participants with MPS lower than 90: 5 TD children and 22 children with ASD. Then we applied linear regression.

In a sample of participants with MPS of 90 or higher, differences in age, ACH, SRS total scores, and all the SRS sub-scales were found to be significant. S3 Table in S1 File presents the characteristics of the restricted sample. Children with ASD are older. They had lower ACH and higher SRS-T scores.

For this sample, we used linear regression to predict a social cognition sub-scale based on MPS or ACH, age, and sex, respectively, in each group. After controlling for age and sex effects, the associations between social cognition sub-scale and MPS or ACH were found to be significant in children with ASD. The results are presented in S4 Table in S1 File.

### Association between K-ABC sub-scales and social cognition in matched participants–CEM

After improving balance using the CEM algorithm, 14 TD children and 9 children with ASD comprised the matched participants. The L1 distance improved from 0.828 to 0.288. Considering CEM weights, logistic regression showed sex differences as not significant [z(1) = -0.54, *p* = .59]. Similarly, regression models with CEM weights showed that the differences in age in months [*t*(23) = 0.56, *p* = .58], MPS score [*t*(23) = -0.31, *p* = .76], and ACH score [*t*(23) = 0.18, *p* = .86] were not significant in the matched sample. S5 Table in S1 File presents characteristics of the matched participants. S1 Fig presents a scatter plot and adjusted prediction using a 95% confidence interval.

After matching, we used linear regression with CEM weights to predict the social cognition sub-scale based on K-ABC scores (i.e., MPS and ACH) and condition (i.e., ASD or TD), as well as their interaction. For the model using MPS, the main effect of the condition [*t*(21) = 3.19, *p* < .01] was found to be significant. The interaction effect was also significant [*t*(21) = -2.68, *p* = .01]. In the model using ACH, the main effect of the condition [*t*(21) = 4.91, *p* < .001] and the interaction effect were found to be significant [*t*(21) = -4.03, *p* < .001]. The results are presented in Table 4.

**Table 4:** Association between the social cognition sub-scale and K-ABC scores in matched participants.

vs. social cognition sub-scale	Coeff.	SE.	β	*t*	*P*>*t*	95% CI		F	*p*	*R*^2^
MPS	-0.22	0.19	-0.19	-1.15	.26	-0.63	0.18	18.16	< .001	0.68
Condition (ASD, 1; TD, 0)	89.2	27.8	3.39	3.19	.00	30.7	147.6			
Condition ×MPS	-0.71	0.27	-2.83	-2.68	.01	-1.3	-0.16			
vs. social cognition sub-scale	Coeff.	SE	β	*t*	*P*>*t*	95% CI		F	*p*	*R*^2^
ACH	0.79	0.16	-0.70	0.49	.63	-0.25	0.41	25.7	< .001	0.71
Condition (ASD, 1; TD, 0)	118.5	13.96	4.51	4.91	< .001	68.0	169.0			
Condition × ACH	-1.0	0.25	-3.95	-4.03	< .001	-1.5	-0.48			

MPS, mental processing scale; ACH, achievement scale; Coeff., regression coefficient; SE, robust standard error; CI, confidence interval; ASD, autism spectrum disorder; TD, typically developed controls.

To elucidate the relation between social cognition and general intelligence, or achievement, we applied simple regression with CEM weights to predict the social cognition sub-scale based on MPS and ACH in each group. For TD children, the associations were not found to be significant. For children with ASD, the association between social cognition sub-scale and MPS was found to be significant [*t*(7) = -5.05, *p* < .01]. Similarly, the association between social cognition sub-scale and ACH was found to be significant [*t*(7) = -4.68, *p* < .01]. As in the case with unmatched participants, higher intelligence was associated with better social cognition.

## Discussion

We found significant association between intelligence and social cognition in children with ASD. The association was not significant for TD children. This result is generally consistent with those of earlier studies exploring the relation between intelligence and social cognition in ASD [21–28] and TD population [22, 25, 28, 30–32]. Therefore, it conceptually replicates and extends earlier findings. One explanation for these associations might be that children with ASD fully capitalize on general intelligence for successful learning in social cognition, although extensive use of intelligence might not be necessary for TD children. Alternatively, autistic impairment in social cognition can be compensated by intelligence despite a continued deficit in basic abilities (e.g., ToM). In other words, children with ASD having higher intelligence can disguise underlying cognitive difficulties. In such cases, the social cognition sub-scale score can be better because SRS is reported by observers. This conjecture is in line with results presented in a recent report of work by Livingston et al. [29]. They compared children with ASD having milder symptoms, as measured by ADOS-G, despite poor ToM performance (i.e., those who disguise the underlying social cognitive difficulties) with those having severer symptoms and poor ToM performance (i.e., those who failed to disguise) and reported that the former group had higher intelligence. Considering the present results, the former group might have been able to disguise impaired ToM using higher intelligence, resulting in better scores in ADOS-G. Another explanation for the present result is that, as Hus and colleagues explained in a report of their study [45], SRS and its sub-scales measure more general cognitive ability than ASD traits specifically. From this perspective, it is noteworthy that the interactive term for the social communication sub-scale is very similar in magnitude to the social cognition sub-scale. The social communication sub-scale might also have been influenced by intelligence, even though it was not significant (S1b Table in S1 File: Interaction between diagnosis and ACH is beta = -.84; *p* = .052 for the social communication sub-scale vs. beta = -.099, *p* = .03 for the social cognition sub-scale). This conjecture is in line with results reported recently by Hus et al. [45]. Based on their large-scale study, they reported a significant relation between higher SRS-Raw total scores and lower non-verbal IQ, in addition to non-ASD-specific behavioral problems and cognitive skills in an ASD population. The present study might extend the results reported by Hus et al. in that the reported relation between SRS-Raw total scores and non-verbal IQ might have been driven by the influence of intelligence on social cognition and social communication sub-scales of SRS.

After restriction to a sample of individuals with MPS of 90 or higher, the association between lower intelligence and higher social cognition sub-scale (i.e., greater difficulties in social cognition) remained significant for children with ASD. Results of our study show a linear association between intelligence and social cognition. This result differs from those found in a study by Rommelse et al., which identified an inverted U-shaped relation (ASD with average IQ outperform in social cognition those with higher and lower IQ, as measured using the Amsterdam Neuropsychological Tasks (ANT) program) [67]. The difference might derive from the age-heterogeneity of participants. We recruited participants in a narrow age range (5–8 years old), although Rommelse et al. did not (6–21 years old). Considering the influence of childhood sociality on cognitive development over time [49], results from age-inhomogeneous populations might not reflect a true relation in childhood.

In our sample, female sex was associated significantly with higher social cognition sub-scale scores, which implies that girls have more difficulties related to social cognition than boys have. Sex differences in autistic symptomatology might be important considering biological and behavioral differences between girls and boys, but few reports have described effects of sex on social cognition in individuals with ASD. In terms of SRS, two reports have described effects of age on the social cognition sub-scale. Torske et al. reported a strong tendency for girls to have higher scores on the social cognition sub-scale [44], but Rodgers et al. reported a minimal effect size (Cohen’s *d* = -0.073) of sex [43]. Reports of other earlier studies using scales other than SRS tend to describe that female subjects with ASD have better social cognition than their male counterparts do. In fact, Isaksson et al. [26] and Muller et al. [30] measured social cognition respectively using RMET and MASC. They reported that female subjects with ASD performed better on those tests than male subjects with ASD did. Other reports have described non-significant results in terms of ToM [24, 27]. One explanation is expected to be that the current findings were driven by the few female subjects in the sample. Alternatively, considering that SRS T-scores are gender-normed but that the other assessments (RMET and MASC) are not, the gender-normed property of SRS T-scores might explain this contradiction: originally, gender-normed T-scores of SRS are developed to correct gender differences observed in normative samples [41]. Recently, however, Hus et al. examined a large sample of 2,368 children with ASD. They reported that gender effects on raw scores in SRS were minimal and not significant [45]. Given that the effects of gender on SRS raw scores are minimal, whereas the gender-normed T-scores are “corrected” for gender differences in the original normative samples, the use of gender-normed scores created an artificial appearance of greater social impairment in girls. Combining non-significant results from other non-gender-normed measures for social cognition, gender-normed SRS T-scores might have exaggerated difficulties in the female children with ASD in our sample.

We also found significant association between age and the social cognition sub-scale score: For TD children, younger age was associated with a higher social cognition sub-scale score (i.e., greater difficulties with social cognition). For children with ASD, however, older age was associated with higher social cognition sub-scale scores. Social cognition tends to improve with age in TD children [68–71], but the SRS is normed so that age effects are not typically significant. In fact, the present result is contrary to those of most large-scale SRS studies of the general population, demonstrating non-significant age-related differences in SRS total scores. For example, Constantino and Todd examined a US population and found no effect of age on SRS raw scores [42]. Similar findings have been reported for German [71], Chinese [72], and Iranian [73] community and clinical samples. In fact, only for a Japanese population did Kamio et al. report a negative effect of age on SRS total scores (younger age was associated with higher SRS total scores) with a modest effect size [74]. The current study of this Japanese population found a similar relation between the age and social cognition sub-scale of SRS. Considering the present result, effects of age on SRS total scores observed by Kamio et al. might be driven by the effects exerted on the social cognition sub-scale. Moreover, the effects of age on the social cognition sub-scale in TD children might be different from country to country (e.g., the relation might be significant only in Japan). Additional research efforts must be undertaken to ascertain whether this relation is universal or culture-dependent.

By contrast, for children with ASD, the present results demonstrated that older age is associated with higher scores on the social cognition sub-scale (i.e., greater difficulties with social cognition). This result is similar to those reported from the study by Hus et al. They reported significant association between higher SRS total scores (i.e., greater difficulties with social reciprocity) and older age in children with ASD [45]. Considering the results presented herein, this relation might be driven by effects of age on the social cognition sub-scale. However, other earlier studies have tended to show significant and positive effects of age on social cognition measured from a single clinical or laboratory observation. For example, older age was associated with better performance on laboratory test measures including ToM [27, 28], social analogical reasoning [69], RMET [26], and facial emotion recognition [21]. In addition to those one-to-one laboratory measures, the scores of Theory of Mind Inventory (ToMI), a parent report measure for theory of mind and social cognition, tend to improve with age [75]. Why is the social cognition sub-scale in SRS biased to attribute more social cognitive ability to younger children with ASD, even though their older counterparts are expected to have higher skills for one-to-one communication (e.g., better performance in ToM, social analogical reasoning, and RMET) and theory of mind? To explain that discrepancy, one must note that our sample comprises children with mean (standard deviation) age of 73.1(10.3) and that children in Japan attend elementary school from the April following their sixth birthday. In fact, in our sample, the older children are elementary school age children. After entering elementary school, education starts to be conducted while they are living in a group. Teachers encourage children to construct close and personal relationships with their classmates. Under these circumstances, latent difficulties of social cognition in children with ASD might begin to surface. Such difficulties might negatively affect parents’ perceptions of social cognition, yielding higher scores on the social cognition sub-scale, and exceeding some advancement in skills for one-to-one communications or theory of mind.

For the current study, we extended earlier findings by examining different associations between intelligence and social cognition in children with ASD versus TD children. Importantly, significant association was found only in children with ASD. For further progress, in light of the developmental aspects of ASD, a longitudinal follow-up study should be conducted to assess the relation between intelligence and social cognition. Change in social cognition over the course of time in relation to intelligence can be expected to provide additional information related to the structure of social cognition in this population. We found a significant effect of intelligence only on the social cognition sub-scale of SRS, although the effect on social communication sub-scale was similar in magnitude. These results indicate a complex relation between intelligence and social reciprocity in children with and without ASD. When using SRS as a measure of social reciprocity, the different influences of intelligence on the respective sub-scales must be considered.

Some limitations are noteworthy. First, we excluded children with known ID. Therefore, these study findings might not be applicable to a typical ASD population that includes comorbid ID. Second, although children having other psychiatric disorders were excluded from the study, the participants still possibly had comorbid disorders that affect cognitive performance because developmental disorders such as attention deficit/hyperactivity disorder and learning disability are difficult to detect at this age. Third, the study participants were recruited from a small region. Considering environmental risk factors for ASD, our results should be generalized with caution. Fourth, we used parent report-based measures only. Considering the different results derived from laboratory and informant-based measures, future research should combine those two measures because they are mutually complementary [76].

## Supporting information

S1 Data (DTA)

S2 Data (XLS)

S1 Fig (TIFF)

S1 File (DOCX)
