# Supplementary figures and images for: Different associations between intelligence and social cognition in children with and without autism spectrum disorders
Source: PLoS One. 2020 Aug 21;15(8):e0235380. doi: 10.1371/journal.pone.0235380 (PMC7444496; doi:10.1371/journal.pone.0235380)

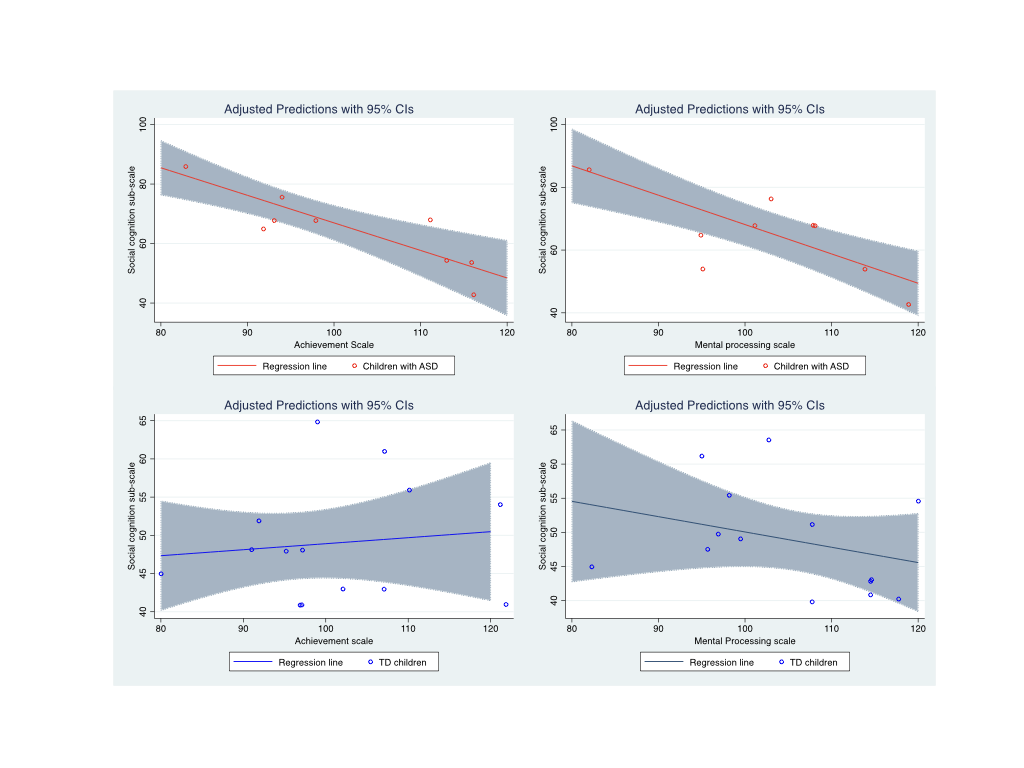

Supplement: S1 Fig — (TIFF) [file pone.0235380.s003.tiff]
