# Supplementary material for: Different associations between intelligence and social cognition in children with and without autism spectrum disorders
Source: PLoS One. 2020 Aug 21;15(8):e0235380. doi: 10.1371/journal.pone.0235380 (PMC7444496; doi:10.1371/journal.pone.0235380)
Supplement: S1 File — (DOCX) [file pone.0235380.s004.docx]

**Sub-tests of K-ABC**

The Sequential Processing Scale comprises three subtests: For *Hand Movements*, the child must imitate the exact sequence of taps on the table made with the fist, palm, or side of the hand as performed by the examiner. For *Number Recall*, the child must repeat, in sequence, a series of numbers presented orally by the examiner. For *Word Order*, the child must point to silhouettes of objects in the same sequence as those objects named by the examiner. The Simultaneous Processing Scale comprises seven subtests: For *Magic Window*, the child must name a picture that is exposed a section at a time through rotation behind a narrow slit. For *Face Recognition*, a picture showing one or two faces is presented briefly. The child must then select the correct face(s) from a subsequently presented group picture. For *Gestalt Closure*, the child shown a partially completed inkblot must then draw and name or describe the drawing. For *Triangles*, a timed task, the child is provided with a set of blue and yellow rubber triangles and is required to duplicate an abstract design presented by the examiner. For *Matrix Analogues*, the child is shown a 2 × 2 visual analogy from which the last element is missing. For all but the earliest items, the child must then select from a set of seven flexible vinyl chips the one which correctly completes the abstract analogy. For *Spatial Memory*, after being shown a set of randomly arranged pictures, the child must recall and indicate their locations on a subsequently presented grid. For *Photo Series*, photographs depicting a sequence of events are placed on the table in random order. The child must order them in their correct sequence by handing them to the examiner.

The Achievement Scale consists of six subtests: For *Expressive Vocabulary*, the child must name the common objects depicted in photographs. For *Faces and Places*, the child must name the fictional character, famous person, or well-known place that is depicted. For *Arithmetic*, the child must count, compute, and demonstrate the child’s understanding of mathematical concepts. For *Riddles*, the child is given attributes, functions, and other general characteristics of a concrete or abstract concept and is asked to infer its name. For *Reading/Decoding*, the child must identify letters and read and pronounce words. For *Reading/Understanding*, the child must act out commands to demonstrate the meaning of the sentence the child has just read [1].

**Supplementary** **Table 1**

Association between SRS sub-scales and K-ABC sub-scales controlling for age and sex

1. Association between social awareness sub-scale and K-ABC sub-scales controlling for age and sex

| vs. social awareness sub-scale | Coeff. | SE | β | *t* | *P*>*t* | 95% CI | |  | F | *p* | *R*^2^ |
| --- | --- | --- | --- | --- | --- | --- | --- | --- | --- | --- | --- |
| MPS | 0.01 | 0.10 | 0.20 | 0.13 | 0.90 | -0.19 | 0.22 |  | 12.49 | < .001 | .37 |
| Condition (ASD, 1; TD, 0) | 21.72 | 13.9 | 0.90 | 1.56 | 0.12 | -5.89 | 49.34 |  |  |  |  |
| Condition × MPS | -0.79 | 0.14 | -0.31 | -0.57 | 0.57 | -0.35 | 0.19 |  |  |  |  |
| Sex | -0.40 | 2.34 | -0.02 | -0.17 | 0.86 | -5.05 | 4.25 |  |  |  |  |
| Age (months) | 0.06 | 0.10 | 0.04 | 0.60 | 0.55 | -0.14 | 0.25 |  |  |  |  |
|  |  |  |  |  |  |  |  |  |  |  |  |
| vs. social awareness sub-scale | Coeff. | SE | β | *t* | *P*>*t* | 95% CI | |  | F | *p* | *R*^2^ |
| ACH | -0.01 | 0.09 | -0.01 | -0.10 | 0.92 | -0.20 | 0.18 |  | 12.34 | < .001 | .38 |
| Condition (ASD, 1; TD, 0) | 20.69 | 13.63 | 0.86 | 1.52 | 0.13 | -6.36 | 47.75 |  |  |  |  |
| Condition × ACH | -0.69 | 0.14 | -0.27 | -0.50 | 0.62 | -0.34 | 0.20 |  |  |  |  |
| Sex | -0.38 | 2.30 | -0.15 | -0.16 | 0.87 | -4.94 | 4.19 |  |  |  |  |
| Age (months) | 0.04 | 0.10 | 0.03 | 0.45 | 0.65 | -0.15 | 0.23 |  |  |  |  |

(b) Association between social communication sub-scale and K-ABC sub-scales controlling for age and sex

| vs. social communication sub-scale | Coeff. | SE | β | *t* | *P*>*t* | 95% CI | |  | F | *p* | *R*^2^ |
| --- | --- | --- | --- | --- | --- | --- | --- | --- | --- | --- | --- |
| MPS | -0.01 | 0.78 | -0.11 | -0.11 | 0.91 | -0.16 | 0.15 |  | 18.69 | < .001 | .53 |
| Condition (ASD, 1; TD, 0) | 40.14 | 13.6 | 1.37 | 2.95 | <0.01 | 13.1 | 67.2 |  |  |  |  |
| Condition × MPS | -0.22 | 0.14 | -0.73 | -1.69 | 0.09 | -0.49 | 0.04 |  |  |  |  |
| Sex | -2.31 | 2.34 | -0.07 | -0.98 | 0.33 | -7.0 | 2.34 |  |  |  |  |
| Age (months) | 0.20 | 0.12 | 0.12 | 1.63 | 0.11 | -0.04 | 0.44 |  |  |  |  |
|  |  |  |  |  |  |  |  |  |  |  |  |
| vs. social communication sub-scale | Coeff. | SE | β | *t* | *P*>*t* | 95% CI | |  | F | *p* | *R*^2^ |
| ACH | -0.03 | 0.05 | -0.03 | -0.54 | 0.59 | -0.14 | 0.08 |  | 19.40 | < .001 | .54 |
| Condition (ASD, 1; TD, 0) | 43.69 | 12.92 | 1.49 | 3.38 | <0.01 | 18.04 | 69.3 |  |  |  |  |
| Condition × ACH | -0.26 | 0.13 | -0.84 | -1.96 | 0.052 | -0.52 | 0.00 |  |  |  |  |
| Sex | -2.41 | 2.12 | -0.08 | -1.13 | 0.26 | -6.66 | 1.83 |  |  |  |  |
| Age (months) | 0.14 | 0.11 | 0.09 | 1.28 | 0.20 | -0.08 | 0.37 |  |  |  |  |

(c) Association between social motivation sub-scale and K-ABC sub-scales controlling for age and sex

| vs. social motivation sub-scale | Coeff. | SE | β | *t* | *P*>*t* | 95% CI | |  | F | *p* | *R*^2^ |
| --- | --- | --- | --- | --- | --- | --- | --- | --- | --- | --- | --- |
| MPS | -0.01 | 0.01 | -0.02 | -0.17 | 0.87 | -0.18 | 0.15 |  | 7.81 | < .001 | .28 |
| Condition (ASD, 1; TD, 0) | 15.2 | 14.11 | 0.60 | 1.08 | 0.28 | -12.8 | 43.3 |  |  |  |  |
| Condition × MPS | -0.04 | 0.15 | -0.13 | -0.25 | 0.81 | -0.33 | 0.26 |  |  |  |  |
| Sex | -3.22 | 2.61 | -0.11 | -1.23 | 0.22 | -8.41 | 2.00 |  |  |  |  |
| Age (months) | 0.18 | 0.14 | 0.13 | 1.28 | 0.20 | -0.10 | 0.46 |  |  |  |  |
|  |  |  |  |  |  |  |  |  |  |  |  |
| vs. social motivation sub-scale | Coeff. | SE | β | *t* | *P*>*t* | 95% CI | |  | F | *p* | *R*^2^ |
| ACH | -0.02 | 0.07 | -0.03 | -0.31 | 0.76 | -0.16 | 0.11 |  | 8.06 | < .001 | 0.28 |
| Condition (ASD, 1; TD, 0) | 15.11 | 13.19 | 0.60 | 1.15 | 0.25 | -11.1 | 41.3 |  |  |  |  |
| Condition × ACH | -0.03 | 0.14 | -0.13 | -0.24 | 0.81 | -0.32 | 0.25 |  |  |  |  |
| Sex | -3.22 | 2.53 | -0.12 | -1.27 | 0.21 | -8.25 | 1.80 |  |  |  |  |
| Age (months) | 0.17 | 0.14 | 0.12 | 1.26 | 0.21 | -0.10 | 0.44 |  |  |  |  |

MPS, mental processing scale; ACH, achievement scale; Coeff, regression coefficient; SE, robust standard error; CI, confidence interval; ASD, autism spectrum disorder; TD, typically developed controls

**Supplementary** **Table 2**

Association between social cognition sub-scale and K-ABC scores

| vs. social cognition sub-scale | Coeff. | SE | β | *t* | *P*>*t* | 95% CI | | F | *p* | *R*^2^ |
| --- | --- | --- | --- | --- | --- | --- | --- | --- | --- | --- |
| MPS | 0.03 | 0.09 | 0.03 | 0.30 | 0.77 | -0.15 | 0.20 | 32.97 | < .001 | .51 |
| Condition (ASD, 1; TD, 0) | 46.73 | 13.61 | 1.60 | 3.43 | 0.00 | 19.73 | 73.74 |  |  |  |
| Condition ×MPS | -0.29 | 0.14 | -0.93 | -2.09 | 0.04 | -0.56 | -0.01 |  |  |  |
|  |  |  |  |  |  |  |  |  |  |  |
| vs. social cognition sub-scale | Coeff. | SE | β | *t* | *P*>*t* | 95% CI | | F | *p* | *R*^2^ |
| ACH | -0.02 | 0.08 | -0.03 | -0.32 | 0.75 | -0.18 | 0.13 | 35.26 | < .001 | .52 |
| Condition (ASD, 1; TD, 0) | 46.93 | 13.96 | 1.60 | 3.36 | 0.00 | 19.23 | 74.63 |  |  |  |
| Condition × ACH | -0.30 | 0.15 | -0.96 | -2.03 | 0.04 | -0.58 | -0.01 |  |  |  |

MPS, mental processing scale; ACH, achievement scale; Coeff.., regression coefficient; SE, robust standard error; CI, confidence interval; ASD, autism spectrum disorder; TD, typically developed controls

**Supplementary Table 3**

Characteristics of participants with MPS of 90 or higher

|  | TD | ASD | χ2 | *t* | *p* |
| --- | --- | --- | --- | --- | --- |
| *n* | 51 | 24 |  |  |  |
| Sex (% Male)^†^ | 65% | 71% | 0.28 |  | .60 |
| Age in months^‡^ | 68.5 (6.8) | 73.6 (9.7) |  | -2.61 | .01 |
|  |  |  |  |  |  |
| K-ABC scores |  |  |  |  |  |
| Mental Processing scale^‡^ | 109.7 (12.1) | 104.2 (14.0) |  | 1.76 | .08 |
| Achievement scale^‡^ | 105.7 (13.6) | 98.5 (15.4) |  | 2.07 | .04 |
|  |  |  |  |  |  |
| SRS-T scores |  |  |  |  |  |
| Total^‡^ | 46.6 (7.0) | 69.6 (14.1) |  | -9.44 | < .001 |
| Social awareness^‡^ | 47.5 (9.6) | 62.8 (10.6) |  | -6.23 | < .001 |
| Social cognition^‡^ | 49.6 (8.2) | 68.4 (14.1) |  | -7.31 | < .001 |
| Social communication^‡^ | 45.4 (7.0) | 65.7 (12.6) |  | -8.90 | < .001 |
| Social motivation^‡^ | 50.0 (6.7) | 65.0 (16.7) |  | -5.61 | < .001 |
| Autistic mannerisms^‡^ | 44.6 (5.2) | 71.9 (17.9) |  | -10.0 | < .001 |

Numbers are mean (standard deviation) or counts.

‡ Student *t*-test

† Chi-square test

**Supplementary** **Table 4**

Association between social cognition and K-ABC sub-scales for respective groups controlling for age and sex in a sample with MPSs of 90 or higher

| vs. social cognition sub-scale | Coeff. | SE | *t* | *P*>*t* | 95% CI | |  | F | *p* | *R*^2^ |
| --- | --- | --- | --- | --- | --- | --- | --- | --- | --- | --- |
| TD |  |  |  |  |  |  |  |  |  |  |
| MPS | 0.04 | 0.11 | 0.35 | 0.73 | -0.20 | 0.28 |  | 1.08 | .37 | .04 |
| Sex | 0.92 | 2.74 | 0.34 | 0.73 | -4.59 | 6.45 |  |  |  |  |
| Age (months) | -0.20 | 0.12 | -1.62 | 0.11 | -0.45 | -0.05 |  |  |  |  |
|  |  |  |  |  |  |  |  |  |  |  |
| ACH | -0.01 | 0.09 | -0.15 | 0.88 | -0.20 | 0.17 |  | 1.01 | .40 | .03 |
| Sex | 1.04 | 2.7 | 0.38 | 0.71 | -4.46 | 6.53 |  |  |  |  |
| Age (months) | -0.20 | 0.12 | -1.68 | 0.10 | -0.46 | 0.04 |  |  |  |  |
| vs. social cognition sub-scale | Coeff. | SE | *t* | *P*>*t* | 95% CI | |  | F | *p* | *R*^2^ |
| ASD |  |  |  |  |  | |  |  |  |  |
| MPS | -0.54 | 0.20 | -2.69 | 0.01 | -0.96 | -0.12 |  | 7.93 | .001 | .45 |
| Sex | -15.1 | 4.84 | -3.12 | 0.01 | -25.2 | -4.98 |  |  |  |  |
| Age (months) | 0.46 | 0.35 | 1.33 | 0.20 | -0.26 | 1.18 |  |  |  |  |
|  |  |  |  |  |  |  |  |  |  |  |
| ACH | -0.36 | 0.12 | -2.90 | <0.01 | -0.62 | -0.10 |  | 5.89 | .005 | .34 |
| Sex | -12.8 | 6.11 | -2.09 | 0.049 | -25.5 | -0.13 |  |  |  |  |
| Age (months) | 0.44 | 0.36 | 1.25 | 0.22 | -0.29 | 1.19 |  |  |  |  |

MPS, mental processing scale; ACH, achievement scale; Coeff., regression coefficient; SE, robust standard error; CI, confidence interval; ASD, autism spectrum disorder; TD, typically developed controls**Supplementary Table 5**

|  | TD | ASD | *z* | *t* | *p*^‡^ |
| --- | --- | --- | --- | --- | --- |
| *n* | 14 | 9 |  |  |  |
| Sex (% male)^†^ | 92% | 89% | -0.54 |  | .59 |
| Age in Months^‡^ | 69.3 (6.4) | 70.3 (6.5) |  | 0.56 | .58 |
|  |  |  |  |  |  |
| K-ABC scores |  |  |  |  |  |
| Mental Processing scale^‡^ | 104.7 (10.9) | 102.8 (11.2) |  | -0.31 | .76 |
| Achievement scale^‡^ | 101.2 (11.5) | 101.8 (12.3) |  | 0.18 | .86 |

Characteristics of the matched participants

Numbers are mean (standard deviation) or counts.

‡ considering CEM weights

K-ABC, Kaufman Assessment Battery for Children; SRS, Social Responsiveness ScaleSupplementary Figure 1

Adjusted predictions with 95% confidence interval and scatter plots.

We predicted marginal means of social cognition sub-scale based on the fitted models with CEM weights [2].

ASD, autism spectrum disorder; TD children, typically developed children

***References***

1. Kaufman AS, O’Neal MR, Avant AH, Long SW. Review Article: Introduction to the Kaufman Assessment Battery for Children (K-ABC) for Pediatric Neuroclinicians. Journal of Child Neurology. 1987. doi:10.1177/088307388700200102

2. Graubard BI, Korn EL. Predictive margins with survey data. Biometrics. 1999. doi:10.1111/j.0006-341X.1999.00652.x
